# Radiological Patterns of Brain Metastases in Breast Cancer Patients: A Subproject of the German Brain Metastases in Breast Cancer (BMBC) Registry

**DOI:** 10.3390/ijms17101615

**Published:** 2016-09-23

**Authors:** Elena Laakmann, Isabell Witzel, Verena Scriba, Ulrich Grzyska, Christine zu Eulenburg, Nicole Burchardi, Tobias Hesse, Florian Würschmidt, Tanja Fehm, Volker Möbus, Gunter von Minckwitz, Sibylle Loibl, Tjoung-Won Park-Simon, Volkmar Mueller

**Affiliations:** 1 Department of Gynecology, University Medical Center Hamburg-Eppendorf, Hamburg 20246, Germany; e.laakmann@uke.de (E.L.); i.witzel@uke.de (I.W.); verena.scriba@stud.uke.uni-hamburg.de (V.S.); 2 Department of Neuroradiology, University Medical Center Hamburg-Eppendorf, Hamburg 20246, Germany; grzyska@uke.de; 3 Department of Medical Biometry and Epidemiology, University Medical Center Hamburg-Eppendorf, Hamburg 20246, Germany; c.h.zu.eulenburg@umcg.nl; 4 Department for Epidemiology, University Medical Center Groningen, Groningen 9700, The Netherlands; 5 German Breast Group GmbH, Neu-Isenburg 63263, Germany; Nicole.Burchardi@gbg.de (N.B.); Gunter.vonMinckwitz@gbg.de (G.v.M.); Sibylle.Loibl@gbg.de (S.L.); 6 Department of Gynecology, Agaplesion Diakonie-Clinic Rotenburg, Rotenburg 27356, Germany; Hesse@diako-online.de; 7 Department of Radiotherapy, Radiology Allianz, Hamburg 22767, Germany; florian.wuerschmidt@radiologische-allianz.de; 8 Translational Research Board of the Arbeitsgemeinschaft für Gynäkologische Onkologie (AGO-Trafo), Duesseldorf 40225, Germany; Tanja.Fehm@med.uni-duesseldorf.de; 9 Breast Study Group of the Arbeitsgemeinschaft für Gynäkologische Onkologie (AGO-B), Frankfurt 65929, Germany; Volker.Moebus@KlinikumFrankfurt.de; 10 Department of Gynecology, Hannover Medical School, Hannover 30625, Germany; Park-Simon.Tjoung-Won@mh-hannover.de

**Keywords:** radiological, pattern, brain metastases, breast cancer

## Abstract

Evidence about distribution patterns of brain metastases with regard to breast cancer subtypes and its influence on the prognosis of patients is insufficient. Clinical data, cranial computed tomography (CT) and magnetic resonance imaging (MRI) scans of 300 breast cancer patients with brain metastases (BMs) were collected retrospectively in four centers participating in the Brain Metastases in Breast Cancer Registry (BMBC) in Germany. Patients with positive estrogen (ER), progesterone (PR), or human epidermal growth factor receptor 2 (HER2) statuses, had a significantly lower number of BMs at diagnosis. Concerning the treatment mode, HER2-positive patients treated with trastuzumab before the diagnosis of BMs showed a lower number of intracranial metastases (*p* < 0.001). Patients with a HER2-positive tumor-subtype developed cerebellar metastases more often compared with HER2-negative patients (59.8% vs. 44.5%, *p* = 0.021), whereas patients with triple-negative primary tumors had leptomeningeal disease more often (31.4% vs. 18.3%, *p* = 0.038). The localization of Brain metastases (BMs) was associated with prognosis: patients with leptomeningeal disease had shorter survival compared with patients without signs of leptomeningeal disease (median survival 3 vs. 5 months, *p* = 0.025). A shorter survival could also be observed in the patients with metastases in the occipital lobe (median survival 3 vs. 5 months, *p* = 0.012). Our findings suggest a different tumor cell homing to different brain regions depending on subtype and treatment.

## 1. Introduction

Brain metastases (BMs) have recently become a major clinical challenge in the management of patients with metastatic breast cancer. Limited data is available about evidence-based treatment strategies of this metastatic entity, and little is known about the biology of the disease [1]. Most of the published data is based on the evaluation of BMs in patient cohorts with different primary tumors, including patients with lung cancer and malignant melanoma. These results cannot be completely transferred on breast cancer patients with BMs: the tumor biology as well as modern therapies distinguish these tumors from other entities.

Several factors for an increased risk of BMs have been identified in breast cancer. Younger patients, poorly differentiated tumors (high grade) and hormone receptor-negative status have been associated with increased BM risk. In HER2-positive and TNBC patients with metastatic breast cancer, incidence rates up to 30%–40% were described [1,2,3]. Although risk factors that are associated with BM formation have been identified, little is known about patterns of BMs depending on molecular subtypes or previous therapies. The biologic characteristics and clinical behavior differ between breast cancer subtypes. For example, patients with BMs from HER2-positive breast cancer live longer in comparison to HER2-negative patients [4]. This is probably due to improved treatment options in this group. Nevertheless, many of these patients develop BMs and the influence of HER2 expression as well as systemic therapies on tumor cell dissemination into the brain, and the growth of tumor cells within the particular microenvironment of the brain is barely understood.

The aim of this analysis was therefore to evaluate the patterns of BMs in different breast cancer subtypes, to estimate an association between BC therapy mode and BM patterns, and to evaluate survival times with regard to BM patterns. The data can provide a background for the prediction of the distribution of BMs in breast cancer patients and support further research concerning prevention, early detection, and treatment of the BMs of breast cancer.

## 2. Results

### 2.1. Patients’ Characteristics

300 patients with breast cancer and brain metastases were available for the evaluation. The median age of the patients at the time of the diagnosis of BMs was 56 years (range: 27–89 years). In 87.3% of the cases (*n* = 262), the cerebral metastases were diagnosed with MRI; in 12.7% of patients (*n* = 38), they were diagnosed with CT. Median time to the development of BMs from first diagnosis of breast cancer was 39 months (range: 0–415) and 13 months (range: 0–131) from the first diagnosis of metastatic disease.

Regarding the number of BMs, 35.0% of the patients (*n* = 105) had one BM, 15.0% (*n* = 45) had two BMs, and 41.0% (*n* = 123) had three or more BMs. Exactly nine percent of the patients had meningeal or spinal metastases as the only BM localization. A total of 11.4% of the patients had parenchymal BMs and leptomeningeal disease simultaneously. Fifty-four percent of primary tumors were hormone receptor-positive (*n* = 141), 44% (*n* = 102) were HER2-positive, and 17% (*n* = 51) were triple-negative. A pathological report of brain metastases was available for 135 patients who received surgical resection of their brain metastases.

During the follow-up period, 58.3% of the patients (*n* = 175) died. Median survival time after BM diagnosis was four months (range: 0–91 months). A detailed summary of patients’ characteristics and therapies is listed in Table 1.

### 2.2. Brain Metastases Patterns and Tumor Subtype

In our evaluation, we could show that patients with positive estrogen, progesterone, or HER2 statuses of the primary breast cancer tumor had a significantly lower number of BMs, also in specific brain regions. ER-negative patients had more BMs than ER-positive patients (mean: 15 vs. 7), and this was also the case for PR-negativity compared with positivity (15 vs. 7) and for HER2-negative compared with HER2-positive patients (15 vs. 8) (*p* < 0.001 each, Table 2).

**Figure 1:**
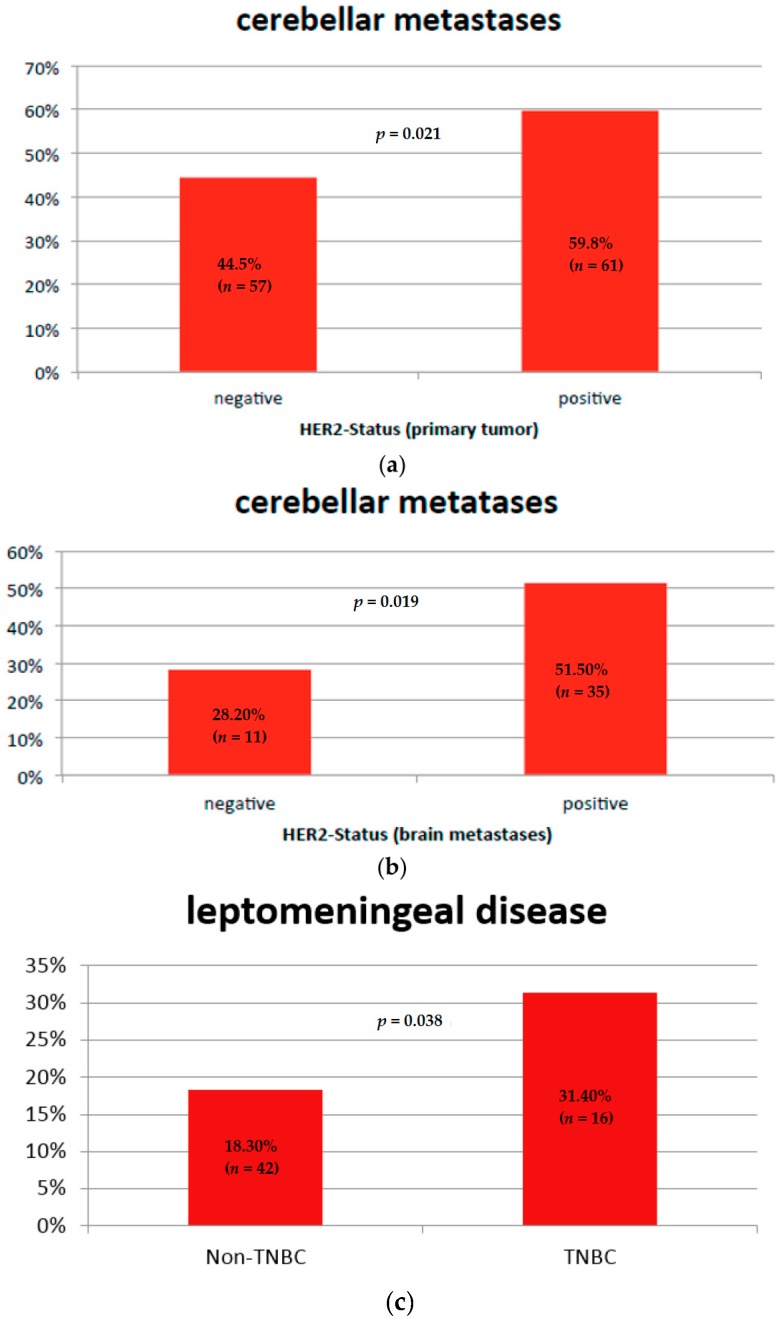
Association between BM patterns and subtypes: (**a**) HER2-status of the primary breast tumor and cerebellar metastases; (**b**) HER2-status (brain metastases) and cerebellar metastases; (**c**) Triple-negative BC and leptomeningeal disease.

Regarding patterns of BMs, patients with HER2-positive primary tumors, HER2-positive BMs, or both had cerebellar metastases more often in comparison with patients with HER2-negative tumors (59.8% vs. 44.5%, *p* = 0.021, Figure 1a,b).

A further correlation could be detected between hippocampal metastases (*n* = 15) and tumor subtypes. Patients with ER-positive or PR-positive tumor biology, or a combination thereof, had a lower incidence of hippocampal metastases compared with patients that were ER-negative, PR-negative, or a combination thereof (2.8% ER-pos. vs. 8.3% ER-neg., *p* = 0.05; 1.6% PR-pos. vs. 9% PR-neg., *p* = 0.009). No significant differences could be detected for patients with HER2-positive or triple-negative BC.

#### Leptomeningeal Disease and Tumor Subtype

Patients with triple-negative breast cancer had leptomeningeal involvement more often (pachymeninx and leptomeninx), diagnosed by imaging (31.4% vs. 18.3%, *p* = 0.038, Figure 1c).

No significant correlation could be detected for patients with other BC subtypes.

### 2.3. Brain Metastases Patterns and Systemic Therapy

In our cohort, 44% (*n* = 102) of patients had HER2-positive primary tumors, and 85.9% (*n* = 85) had received HER2-targeting agents before the diagnosis of BMs: *n* = 83 trastuzumab, *n* = 10 lapatinib, and *n* = 7 pertuzumab.

Our analysis indicates that HER2-positive patients treated with trastuzumab before the diagnosis of BMs had a lower number of BMs compared with HER2-positive patients without previous HER2-targeted therapy (mean 8 vs. 11, *p* < 0.001).

#### Leptomeningeal Disease and Systemic Therapy

No clinically significant correlation could be detected between the development of leptomeningeal disease and the systemic therapy of breast cancer.

### 2.4. Brain Metastases Patterns and Patient Outcome

**Figure 2:**
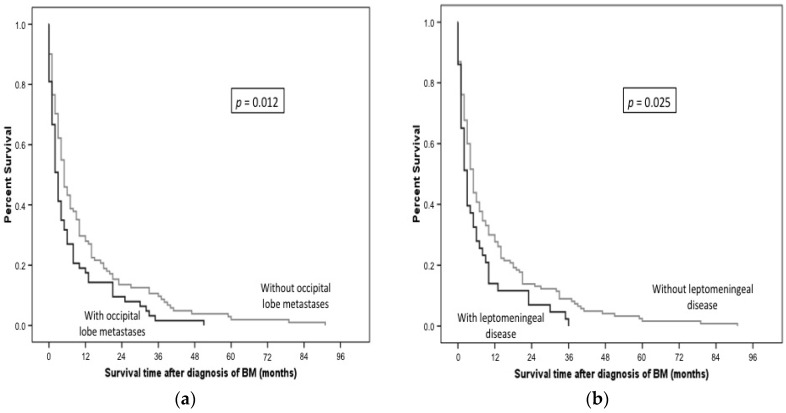
Overall survival depending on BM localization (months). (**a**) Occipital lobe metastases and survival of the patients; (**b**) Leptomeningeal disease and survival of the patients.

Median survival time after the diagnosis of BMs was four months (range: 0–91) in our cohort. A correlation with survival could be shown for patients with metastases in the occipital lobe: patients with occipital lobe metastases (*n* = 63) had a median survival of three months compared with five months for patients without metastases in the occipital lobe (*n* = 111) (*p* = 0.012, Figure 2a).

No correlation was detected between other parenchymal BM localizations and survival of the patients.

#### Leptomeningeal Disease and Patient Outcome

Patients with leptomeningeal disease (*n* = 43) had a shorter survival compared with patients without leptomeningeal disease (*n* = 130) (median survival 3 vs. 5 months, *p* = 0.025, Figure 2b).

In multivariate analyses, brain irradiation, neurosurgery, and positive HER2-status of the primary tumor were associated with longer survival after the diagnosis of BMs. Age at diagnosis of BMs was inversely associated with survival: a higher age was associated with an impaired prognosis of patients (Table 3).

## 3. Discussion

This retrospective study of BM patterns in 300 breast cancer patients showed that there may be a link between the histological subtype of the primary tumor, previous therapies and localization, and the number of BMs. In addition, we give evidence that there may be an association between the locations of BMs with survival.

To our knowledge, only a few smaller studies have been published examining the association between the radiological BM patterns and breast cancer subtype.

Lekanidi et al. evaluated 60 breast cancer patients with BMs and showed that ER-negative HER2-positive patients were more likely to present with a larger number of lesions, more brain stem or occipital metastases, and hydrocephalus [5]. In our analysis of a large cohort of 300 patients, we could confirm that estrogen-negative patients had a higher number of BMs in comparison with ER-positive patients.

In line with our results, Niwinska et al. [6] showed that patients with a triple-negative tumor-subtype had a higher probability to metastasize to the leptomeninges.

We could demonstrate that HER2-positive patients were more likely to metastasize into the cerebellum, which was also suggested by Tomasevic in a preliminary report on 18 patients [7].

In our cohort of breast cancer patients with BMs, we could show that the number of BMs was also correlated with previous systemic therapy. The analysis of the correlation between BM patterns and previous systemic therapy showed that HER2-positive patients treated with trastuzumab before the diagnosis of BMs had a lower number of BMs in comparison with patients without HER2-targeted therapy. A protective role of the HER2-targeted therapy concerning the BMs was also suggested by the findings of Duchnowska et al., where no trastuzumab administration in the metastatic setting was an independent variable in the development of early BMs [8].

Our finding indicating a lower number of BMs in HER2-positive patients treated with the HER2-targeting compound trastuzumab could be of clinical relevance since it might be a sign of its effect in the brain. This has been a controversial issue. Some published data suggests that trastuzumab only penetrates to a small degree via the blood–brain barrier [9], whereas other investigations indicate increased trastuzumab levels in cerebrospinal fluid under conditions of an impaired blood–brain barrier such as BMs [10].

The finding about specific metastases patterns of HER2-positive (cerebellum) and triple-negative patients (leptomeninges) could have several implications. First, in clinical care, physicians could pay attention to clinical symptoms associated with each of these metastatic localizations. Moreover, the different behavior of biologic subtypes, which seems to be also influenced by systemic therapy, should lead to experimental research that might help to understand the mechanisms underlying such behavior and finally to improve strategies for the prevention and treatment of BMs.

We initially hypothesized that the temporal lobe and hippocampus were never involved in the metastatic process to the brain in BC patients, but we could not confirm this hypothesis, as we found that each region in the brain might be involved in the metastases formation. However, the rate of hippocampal metastases in our cohort was very low (*n* = 15 of 300 patients); therefore, radiotherapy sparing the hippocampal regions to avoid neurologic impairment might be discussed with the patient. Our findings go with the results of a study by Hong et al., where a low rate of metastases in the hippocampal region was seen in an analysis of 77 melanoma patients [11].

Concerning the association between BM patterns and prognosis of the patients, several reports have been published. In our investigation, leptomeningeal disease and occipital lobe metastases were associated with poor prognosis. Jo et al. and Hyun et al. also described a poor prognosis of patients with leptomeningeal disease [12,13]. To our knowledge, we are the first to give data about the impact of other specific BM localizations on the survival of BC patients.

The strength of our analysis is the large sample size of 300 patients with BMs of breast cancer. However, the evaluation of the scans was done retrospectively, and not all patients had received an MRI scan of the brain, which does not allow a complete comparison. In addition, only 45% had received resection of the BMs. We could not take into consideration all cases of possible receptor changes [14,15] from primary to metastatic sites. The missing information concerning the biological characteristics of some patients results from a retrospective patient cohort treated several years ago. However, the inclusion of these patients provided inter alia an opportunity to analyze a historic cohort of HER2-positive patients without HER2-targeted therapy.

The evaluation of the presented issue in a prospective manner could provide further evidence about the association of breast cancer subtypes, treatment and radiological BM patterns.

Taken together, we have added new data on the development of BMs depending on tumor subtype and preceding therapies. The different behaviors of biologic subtypes, which seems to be additionally dependent on systemic therapy, could lead to research in experimental models that might help to improve the understanding of mechanisms underlying such behavior and therefore to improve strategies for the prevention and treatment of BMs.

## 4. Materials and Methods

The clinical data of 300 patients with BMs of breast cancer (first diagnosis of breast cancer BMs between 1986 and 2016) was documented retrospectively and also prospectively in the Brain Metastases in Breast Cancer (BMBC) Network in Germany [16]. The BMBC registry is approved by the ethics committee Hessen (processing number FF42/2013). The data for this subanalysis was collected in four participating German centers: University Medical Center Hamburg-Eppendorf, Hannover Medical School, the Oncologic Department of the Agaplesion Diakonie-Clinic Rotenburg, and an outpatient imaging center. The assessment of the cerebral computer tomography (CT) and magnetic resonance imaging (MRI) at the timepoint of the diagnosis of BMs was performed in all cases with available image material (*n* = 271) by one investigator and double-checked in 10% of cases by an independent investigator. For 29 patients, only the written reports were taken into consideration, as image material was no longer available. The affected brain regions and the number of the metastases in each brain area were analyzed. The last follow-up was performed in January 2016.

HER2 positivity was defined as either immunohistochemical staining of 3+ or a positive FISH-result.

The association between the BM localization and the tumor subtype or therapy mode was calculated with a chi-square test. In cases with surgical resection of BMs and available pathology reports of the BMs, the association between BM localization, subtype of the primary, and the brain tumor was analyzed.

Influence of tumor subtype as well as the preceded therapy on the number of BMs was estimated with Poisson-regression analysis.

Survival analysis was performed with the univariate log-rank test.

The multivariate cox-regression analysis took into consideration the histological subtype of the primary tumor, the number of BMs, BM treatment, visceral metastases, and the age of BM diagnosis.

Survival was defined as the interval between diagnosis of BMs and the date of death.

Statistical analyses were performed with the SPSS Statistics Software, Version 21 (IBM, SPSS Inc., Chicago, IL, USA). Five percent was considered significant.

## Figures and Tables

**Table 1:** Patients’ characteristics.

Patient’s Characteristic		Median or Number	Range or Percent
Age at diagnosis of breast cancer in years		51 (median)	Range 23–89
Age at diagnosis of BMs in years		56 (median)	Range 27–89
Time to brain metastases from first diagnosis of BC in months		39 (median)	Range 0–415
Survival after BM diagnosis in months		4 (median)	Range 0–91
Estrogen-receptor (*n* = 261, *n* = 39 missing data)	positive	141	54.0%
	negative	120	46.0%
Progesterone-receptor (*n* = 260, *n* = 40 missing data)	positive	125	48.1%
	negative	135	51.9%
HER2-receptor (*n* = 230, *n* = 70 missing data)	positive	102	44.3%
	negative	128	55.7%
Triple-negative primary tumor (*n* = 281, *n* = 19 missing data)	yes	51	18.1%
	no	230	81.9%
Chemotherapy before the diagnosis of BMs (*n* = 267, missing data *n* = 33)	yes	238	89.1%
	no	29	10.9%
Endocrine therapy before the diagnosis of BMs (*n* = 136, missing data *n* = 5)	yes	116	85.3%
	no	20	14.7%
HER2-targeted therapy before the diagnosis of BMs in the subgroup of patients with a HER2-positive BC (*n* = 99, missing data *n* = 3)	yes	85	85.9%
	no	14	14.1%

**Table 2:** Number of the brain metastases depending on BC subtype.

Receptor Status		BM Number (Mean, SD)	*p*-Value
Estrogen receptor status	positive	7.19 (0.24)	<0.001
	negative	15.26 (0.37)	
Progesterone receptor status	positive	6.95 (0.25)	<0.001
	negative	14.56 (0.34)	
HER2 status	positive	8.24 (0.29)	<0.001
	negative	15.44 (0.38)	

**Table 3:** Association between patient characteristics and survival (multivariate analysis).

Variable	Hazard Ratio	*p*-Value	95% Confidence Interval
HER2-negativity	1.685	0.008	1.144–2.480
Older age at diagnosis of BMs	1.016	0.037	1.001–1.031
No radiotherapy of the brain	3.297	<0.001	2.042–5.322
No neurosurgery	1.981	0.002	1.297–3.026
